# Sustainable aging at work: a process framework for capability, choice and work–retirement transitions in the Nordic–Baltic region

**DOI:** 10.3389/fpsyg.2026.1988720

**Published:** 2026-10-21

**Authors:** Ieva Reine, Kolbeinn H. Stefánsson, Raimonda Sadauskaitė, Antanas Kairys, Olga Zamalijeva, Madara Miķelsone, Olga Rajevska, Luule Sakkeus, Halldór S. Guðmundsson

**Affiliations:** 1 Department of Public Health and Caring Sciences, Uppsala University, Uppsala, Sweden; 2 Statistics Unit, Rīga Stradiņš University, Riga, Latvia; 3 Faculty of Social Work, University of Iceland, Reykjavík, Iceland; 4 Institute of Psychology, Vilnius University, Vilnius, Lithuania; 5 School of Governance, Law and Society, Estonian Institute for Population Studies, Tallinn University, Tallinn, Estonia

**Keywords:** ageism, aging at work, Baltic states, digitalization, job quality, Nordic countries, older workers, retirement

## Abstract

Population aging has intensified interest in extending working lives, yet employment at older ages is not in itself evidence of sustainable aging at work. This article develops a Sustainable Aging-at-Work Alignment Framework that conceptualizes later-life work and retirement as a dynamic process shaped by the interaction of individual capabilities and preferences, workplace demands and resources, and institutional opportunities and constraints. Alignment or misalignment among these conditions influences transition mechanisms through which people may be pushed or pulled toward retirement, choose to stay, remain because alternatives are constrained, or move toward valued non-work goals. These mechanisms give rise to different work–retirement trajectories whose sustainability is evaluated over time in relation to health, psychological wellbeing, autonomy and meaningful choice. The framework draws on successful aging at work, person–environment fit, job demands–resources, work ability, sustainable employability, capability and retirement-transition perspectives, while linking them to the substantive availability of retirement alternatives. Ageism operates across individual, workplace and institutional levels and can shape both perceived capabilities and objective opportunities. The Nordic–Baltic region provides a theoretically informative setting because common demographic pressures coexist with substantial differences in pension adequacy, retirement flexibility, labor-market institutions and late-career opportunities. Five propositions address cross-level alignment, work ability and job demands, digital adaptation, unequal access to flexibility, and the translation of retirement intentions into voluntary or constrained transitions. The framework shifts attention from employment duration alone to the processes, degree of choice and longer-term consequences that characterize later-life work and retirement trajectories.

## 1 Introduction

Population aging is reshaping European labor markets. Longer life expectancy, lower fertility and rising old-age dependency ratios have encouraged governments to increase pension ages, restrict early exit routes and promote employment at older ages. These reforms have contributed to a marked rise in older-worker employment across the OECD. Yet the policy objective of extending working lives contains an important conceptual ambiguity: more years in employment are not necessarily better years in employment (Kuitto and Helmdag, 2021; OECD, 2025a; Phillipson, 2019).

Research on aging and work increasingly shows that later-life employment is shaped by more than pension eligibility. Health and work ability, psychosocial and physical working conditions, skills, employer practices, family responsibilities, pension adequacy, age norms and opportunities for flexible work all influence whether people can and want to remain employed (Baxter et al., 2021; Sousa-Ribeiro et al., 2021; Vickerstaff and van der Horst, 2021). This literature increasingly treats successful aging at work as a multidimensional process involving worker resources, work design and social context rather than employment duration alone (Kooij et al., 2020).

The Nordic and Baltic countries provide an unusually informative setting for studying these processes. They are geographically close and economically interconnected, but they differ in welfare-state development, pension design, labor-market institutions, population health, income security and employment patterns. The Nordic countries are often grouped together as high-employment welfare states, while Estonia, Latvia and Lithuania share post-socialist institutional histories. Such broad labels, however, can conceal substantial variation within each sub region, including differences in pension flexibility, employment patterns, job quality and income security (Grødem and Hippe, 2021; Kuitto and Helmdag, 2021).

Existing research has established that later-life employment and retirement are shaped by individual resources, work characteristics and institutional conditions. What remains less well explained is how these conditions combine to produce transitions that may appear similar in employment statistics but differ fundamentally in their sustainability. Continued employment may reflect good health, meaningful work and a preference to remain employed, but the same observed outcome may arise from financial necessity, restricted retirement alternatives or an inability to exit unsuitable work. Conversely, retirement may represent either a preferred transition or an involuntary response to declining health, adverse working conditions or exclusion from employment. A theoretical account of sustainable aging at work therefore needs to distinguish the observed employment transition from the conditions and mechanisms producing it and from the criteria used to evaluate its sustainability.

The principal contribution of this article is a process framework explaining why similar observed late-career employment outcomes may represent very different degrees of sustainability. The Sustainable Aging-at-Work Alignment Framework links multilevel antecedent conditions to alignment/misalignment, transition mechanisms, work–retirement trajectories and subsequent sustainability outcomes. It therefore goes beyond locating determinants at individual, workplace and institutional levels. The Nordic–Baltic region is used as a theoretically informative comparative setting rather than as a separate contribution or country ranking: similar demographic pressures coexist with substantial variation in pension adequacy, retirement flexibility, labor-market institutions, health, adult learning and late-career employment patterns. This heterogeneity permits theoretically relevant contrasts in the opportunities and constraints through which capability and preference are translated into continued work, modified work or retirement.

## 2 Why the Nordic–Baltic region should be analyzed comparatively

The eight Nordic–Baltic countries considered here share demographic pressures but differ in the institutional and labor-market conditions shaping later-life employment. All face population aging and pressures to maintain labor supply, yet the meaning of working longer varies across pension systems, labor markets and welfare arrangements. A high employment rate can reflect good health, attractive jobs and strong demand for experienced workers, but it can also reflect limited pension income or restricted exit options. Conversely, lower employment at older ages may indicate more generous retirement protection, poorer health, weak employer demand or a combination of these factors. Comparative analysis is therefore necessary to distinguish similar employment outcomes that arise through different mechanisms (Kuitto and Helmdag, 2021; Scherger, 2021; Spasova et al., 2025). Table 1 summarizes these contrasts as an analytical profile rather than a country ranking: the country-specific patterns are used to identify different mechanisms through which later-life employment may become more or less sustainable.

**Table 1:** Comparative analytical profile of the Nordic–Baltic countries, grouped by region.

Country	Institutional/work configuration	Transition mechanism to examine	Framework implication
Nordic countries			
Denmark	Collectively negotiated senior flexibility; state pension age 67	Stay vs. push; sectoral access to negotiated flexibility	Institutional flexibility should be more sustainable where workplace implementation and bargaining coverage permit its use.
Finland	Work-ability orientation; cohort-specific pension eligibility	Alignment between changing work ability and job demands	Work-ability/job-demand misalignment should predict less favorable trajectories unless adaptations compensate.
Iceland	Very high late-career employment; flexible work–retirement combinations	Stay vs. stuck cannot be inferred from participation alone	High participation requires evidence on preference, material constraint and perceived choice.
Norway	Flexible pension withdrawal and work–pension combination	Stay/stuck and pull may coexist	Flexible pathways may widen choice unevenly by class, health and occupation.
Sweden	High older-worker participation; flexible retirement; age-inclusive work	Stay vs. push under heterogeneous job quality	Workplace resources and adaptations should condition whether institutional flexibility produces sustainable continuation.
Estonia	High work after pension receipt; flexible work–pension combination	Stay and stuck may coexist	Pension adequacy and material resources should condition the voluntariness of continuation.
Latvia	High work after pension receipt; adequacy concerns	Stuck vs. stay	The same continuation outcome may reflect attachment or financial constraint.
Lithuania	High work after pension receipt; completed pension-age equalization	Stuck/stay and retirement-intention alignment	Material resources and job quality should shape whether continuation is chosen or constrained.
OECD average	External benchmark only	Not a transition type	Benchmarking is descriptive; aggregate employment cannot establish sustainability or voluntariness.

Country configurations are heuristic comparative cases, not validated welfare-regime classifications or rankings. They identify theoretically relevant contrasts for the propositions; observed national averages cannot establish the voluntariness or sustainability of individual trajectories.

OECD evidence illustrates this heterogeneity. In 2024 the OECD average employment rate at ages 60–64 was 55.9%, while Iceland reached 77.2%, the highest rate in the OECD (OECD, 2025a). At the same time, Eurostat 2023 *ad hoc* Labor Force Survey module shows that Estonia and Latvia were among the European countries with the largest proportions of people continuing to work after receiving an old-age pension (Eurostat, 2024). These indicators describe different processes: one measures employment before and around pension age; the other captures behavior after pension receipt. Neither can be treated as a direct measure of sustainable aging at work.

## 3 Sustainable aging-at-work alignment framework

We define sustainable aging at work as a dynamic property of work-retirement trajectories in which individuals can adapt their participation in paid work over time in ways that remain compatible with their evolving capabilities and preferences and preserve meaningful choice between continued work, modified work and retirement. Sustainability is evaluated through the longer-term consequences of these trajectories for health, psychological wellbeing and autonomy rather than through employment duration alone. Continued employment is therefore neither a necessary nor a sufficient condition for sustainable aging at work. This definition distinguishes sustainability outcomes from their antecedents: health and work ability, psychological resources, job quality, workplace practices, material resources and institutional conditions can shape trajectories without being conceptually identical to sustainability itself.

The framework distinguishes four analytically related components (Figure 1). First, antecedent conditions and resources operate across individual, workplace and institutional levels. Individual conditions include physical and mental health, work ability, subjective age, self-efficacy, perceived control, motivation, skills, retirement preferences and proactive adaptation such as job crafting; workplace conditions include psychosocial and physical demands and resources, autonomy, flexibility, age-inclusive practices, idiosyncratic deals, access to learning, digital adaptation and opportunities to modify tasks; institutional and socioeconomic conditions include pension eligibility and adequacy, taxation, employment protection, alternative exit routes and possibilities to combine work and pension. Second, alignment/misalignment captures compatibility among these evolving capabilities, preferences, demands, opportunities and constraints. Third, transition mechanisms translate these configurations into trajectories of continued employment, modified or reduced employment, work–pension combinations and retirement. Fourth, sustainability is evaluated through subsequent trajectories of health, psychological wellbeing, autonomy and meaningful choice.

**Figure 1:**
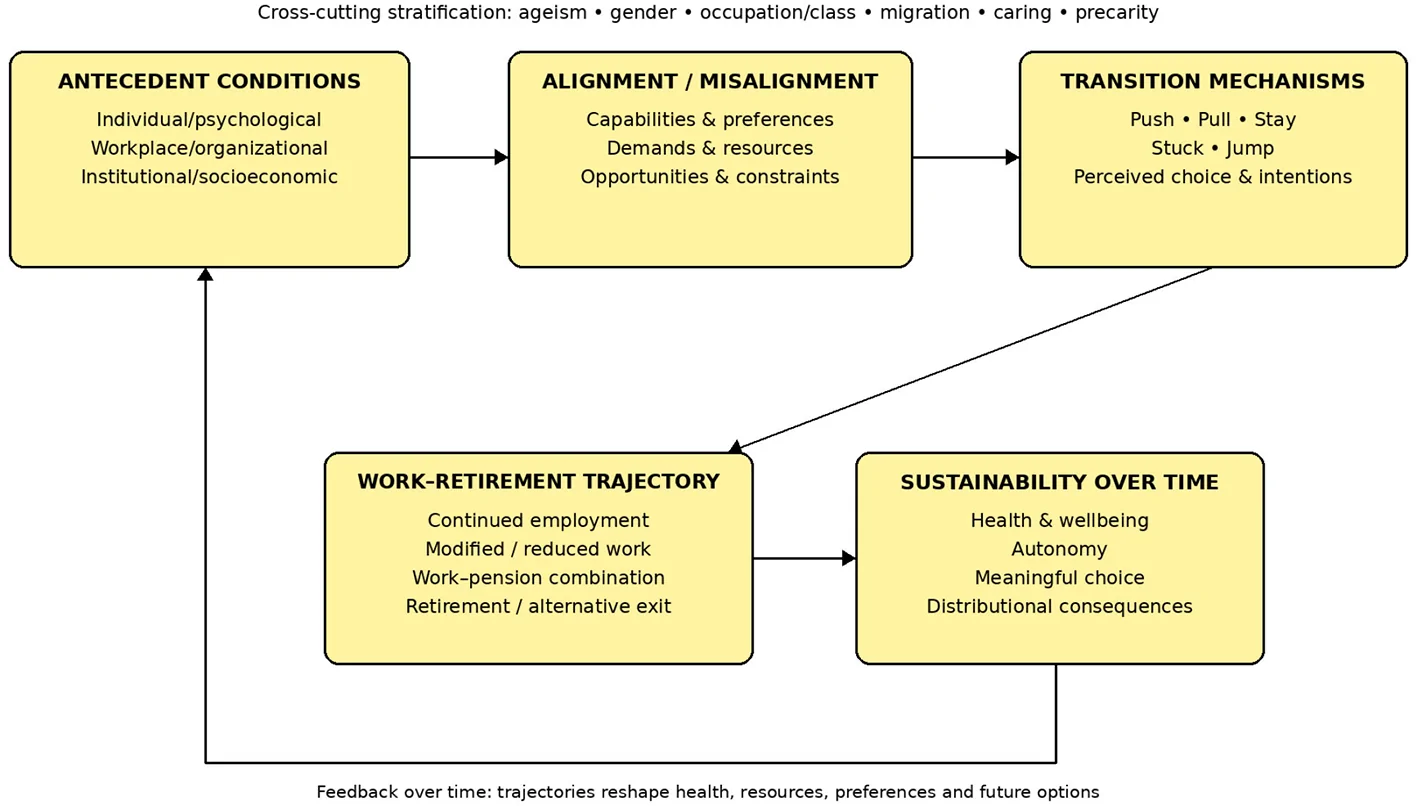
Sustainable Aging-at-Work Alignment Framework: multilevel antecedents are translated through alignment/misalignment and transition mechanisms into work–retirement trajectories whose sustainability is evaluated over time. Ageism and social inequalities operate across the process.

Alignment is the central relational construct of the framework. It refers to the degree of compatibility, at a given point in time, between a worker's evolving capabilities and preferences, job demands and resources, and institutional and socioeconomic opportunities and constraints. Alignment may be objective (for example, functional capacity relative to physical demands or actual eligibility for a preferred pension pathway) and subjective (for example, perceived control, self-efficacy, perceived feasibility and perceived fit with personal goals). It is conceptualized as a cross-level configuration rather than a single score or universal threshold. Misalignment is indicated when consequential demands exceed resources, when preferences and feasible options diverge, or when formally available options cannot be used in practice. Compensation is possible—for example, job redesign can offset declining functional capacity—but is bounded by material and institutional constraints. Empirically, alignment can be tested using multilevel interactions, response-surface approaches for specific fit relations, latent-profile/configurational analyses for patterns of conditions, and longitudinal models of change. Competing operationalizations are a feature to be tested rather than assumed to be equivalent.

Ageism represents a cross-cutting stratifying process rather than a separate level of the framework. Internalized age stereotypes may affect subjective age, self-efficacy, perceived competence, motivation and retirement intentions; direct and indirect workplace age discrimination may shape recruitment, training, promotion, task allocation, recognition, access to flexibility and workplace adaptation; and institutional protections, enforcement and cultural age norms can expand or constrain later-career opportunities. Ageism can therefore alter both objective opportunity structures and subjective appraisals of capability and choice, and it may intersect with gender, occupational class, migration history, caring responsibilities and employment precarity (Vickerstaff and van der Horst, 2021).

### 3.1 Theoretical positioning and distinctiveness

The framework builds on, but is not intended to replace, several established traditions. The successful-aging-at-work model emphasizes adaptive processes through which workers maintain or improve functioning across the working lifespan (Kooij et al., 2020). Person–environment fit focuses on congruence between individual abilities, needs and values and the work environment (Rauvola et al., 2020), while job demands–resources approaches explain how demands and resources influence strain and motivation. Work-ability research focuses more specifically on the balance between functional capacity and work demands. Sustainable-employability research, particularly capability-based formulations, asks whether workers are able and enabled to achieve work outcomes they value while safeguarding health and welfare (van der Klink et al., 2016). Retirement research, in turn, conceptualizes retirement as a process shaped by individual, family, work and sociocultural antecedents rather than a single event (Fisher et al., 2016; Wang and Shultz, 2010), alongside job demands–resources and work-ability perspectives (Bakker and Demerouti, 2007; Ilmarinen, 2009).

Bridge-employment research is especially relevant because work after formal retirement or pension uptake can represent a distinct transition rather than simple continuation of the pre-retirement job. Studies of bridge-employment intentions show that work characteristics, motivational orientations and psychological work ability shape willingness to remain economically active after retirement, while reviews emphasize that bridge employment may serve both financial and psychosocial functions (Alcover and Topa, 2018; Carlstedt et al., 2018). In the present framework, bridge employment is therefore treated as one possible work–retirement trajectory whose sustainability cannot be inferred from participation alone: the same arrangement may reflect valued continuity and choice or material constraint and limited alternatives.

The psychological level is further informed by lifespan-development perspectives. Selection–optimization–compensation emphasizes adaptive allocation of resources as capacities and opportunities change with age, while motivational lifespan theories emphasize control striving, goal adjustment and the regulation of attainable goals. In later careers, self-efficacy, perceived control, subjective age and proactive behaviors such as job crafting or negotiating idiosyncratic deals can therefore influence whether workers adapt the job, revise retirement intentions, or experience declining fit. These mechanisms give psychological content to alignment without assuming that adaptation is solely an individual responsibility; the feasibility of proactive adaptation remains conditioned by organizational opportunity and institutional resources (Baltes and Baltes, 1990; Heckhausen et al., 2010).

The added explanatory value of the present framework lies in connecting these traditions to retirement alternatives and institutional constraints. Alignment is broader than person–job fit because the relevant “environment” includes not only the job but also pension eligibility, income security, household circumstances and the practical availability of alternative trajectories. It is narrower than sustainable employability because the explanandum is specifically the late-career work–retirement process. The framework also separates antecedent resources from transition mechanisms and from the outcomes used to evaluate sustainability, thereby avoiding treating good health or job quality simultaneously as both cause and definition of a sustainable trajectory. The capability perspective is particularly important for the concept of genuine choice: formally available options are not equivalent to substantive opportunities if workers lack the health, resources, workplace accommodation or financial security required to use them (van der Klink et al., 2016).

### 3.2 From alignment to transition mechanisms

To specify how multilevel conditions are translated into behavior, we use push–pull–stay–stuck–jump as a complementary transition vocabulary rather than as an alternative theory of alignment. Push denotes involuntary pressure toward exit, for example through poor health, adverse work or age discrimination; pull denotes attraction toward retirement through accessible pension arrangements or social norms; jump denotes voluntary movement toward valued non-work goals; stay denotes voluntary continuation because work remains attractive or meaningful; and stuck denotes involuntary continuation when retirement is not practically affordable or accessible. These mechanisms can coexist and change over time; they are not mutually exclusive worker types. A person may simultaneously value work and need income, or be attracted to retirement while being pushed by deteriorating conditions. Preferences can also adapt to constraint. The framework therefore treats perceived choice as graded and revisable rather than as a simple voluntary/involuntary dichotomy (Fisher et al., 2016; Wang and Shultz, 2010).

Alignment/misalignment conditions the relative salience of these transition mechanisms. Alignment between capacity and job demands, meaningful work and adequate resources can support stay processes; deteriorating person–work alignment can strengthen push processes; generous and accessible retirement arrangements can create pull opportunities; attractive post-work projects can support jump; and inadequate pension income can generate stuck continuation. Institutional and workplace conditions therefore affect both the direction of transition and its experiential meaning.

### 3.3 Temporal dynamics and feedback

Sustainable aging at work is explicitly temporal. Distal conditions such as cumulative occupational exposure, education, career history and pension design shape proximal appraisals of work ability, control, financial feasibility and retirement alternatives. Preferences may develop into intentions, intentions into decisions, and decisions into behavior, but each link can be revised as health, household circumstances, job demands or pension eligibility change. Transitions also feed back into subsequent health, wellbeing, income and identity. Continued employment that initially reflects stay may later become stuck; conversely, workplace redesign may convert an unwanted extension of work into an acceptable or preferred arrangement. Sustainability must therefore be assessed across trajectories and consequences rather than at a single employment-status observation.

## 4 Evidence base and comparative strategy

Throughout the comparative analysis, “older workers” is used as a relational rather than a fixed chronological category, reflecting differences in pension ages and institutional retirement thresholds across countries. For empirical indicators, the relevant age range is reported explicitly (e.g., 55–64 or 60–64 years); in the theoretical framework, the term refers more broadly to workers in later career approaching or crossing institutionally relevant retirement thresholds.

This is a theory-building comparative synthesis rather than a systematic review or individual-level causal analysis. The evidence base was assembled purposively to support concept development and comparative case selection. It combines (1) current OECD and Eurostat indicators; (2) official pension and social-insurance information applicable in 2026; (3) landmark and review literature on successful aging at work, person–environment fit, work ability, job demands and resources, sustainable employability/capability, retirement timing and bridge employment; and (4) Nordic–Baltic empirical studies on work quality, retirement intentions, age management, digitalization and inequality. The conceptual literature was identified through targeted searches and backward/forward reference checking around major reviews and theoretical papers, with priority given to peer-reviewed reviews, longitudinal studies and official comparative sources available up to September 2026.

Study and indicator selection was guided by conceptual relevance rather than statistical significance. Contradictory findings were retained where they indicated heterogeneity in mechanisms or populations. Evidence from conference abstracts and non-peer-reviewed reports is identified as such and is not used as the sole basis for a central theoretical claim. National characteristics are described as empirically documented only when supported by official or peer-reviewed sources; otherwise they are explicitly labeled heuristic interpretations. Comparisons include Iceland, Estonia, Latvia, Lithuania, Sweden, Denmark, Finland and Norway, with OECD or EU benchmarks where definitions are comparable. Because indicators differ in age range, denominator and institutional meaning, they are not combined into a composite ranking.

## 5 Comparative evidence: employment, pensions and retirement behavior

### 5.1 Working longer is not a single Nordic–Baltic pattern

Older-worker employment has increased across OECD countries, particularly among women, but employment still declines sharply after age 60. The OECD reports an average employment rate of 55.9% at ages 60–64 in 2024, with Iceland at 77.2%. This places Iceland at one end of the comparative spectrum, but it should not be interpreted as a universal model. The substantive question is what enables people to remain employed and whether the same mechanisms operate across occupational, educational, health and gender groups (OECD, 2025a).

Post-pension employment reveals another pattern. Eurostat reported that 13% of people in the EU continued working after receiving their first old-age pension in 2023. Estonia (54.9%), Latvia (44.2%) and Lithuania (43.7%) had the highest shares in the EU. The denominator is people who received an old-age pension, and the indicator is therefore conceptually different from an age-specific employment rate. Across the EU, the two most frequently reported reasons for continuing were enjoyment of work/being productive (36.3%) and financial necessity (28.6%). The published Eurostat summary does not provide country-specific percentages for each motive, so such percentages are not inferred here. However, the OECD's synthesis of the same European evidence reports that financial reasons predominate among those working beyond retirement in the Baltic states, in contrast to Norway, where enjoyment of work predominates. This strengthens the rationale for distinguishing stay from stuck mechanisms while avoiding unsupported country-level motive estimates (Eurostat, 2024; OECD, 2025b).

Estonian evidence further suggests that continued work at older ages is socially selective and institutionally conditioned. Those who continue working tend to be in better health (Klesment and Leppik, 2012), while low pension replacement rates and other institutional constraints can make continued employment less a matter of unconstrained individual choice (Unt and Saar, 2016). Retirement expectations may also diverge from preferences, with some older workers remaining in employment despite a desire to retire (Lindemann and Unt, 2016).

A broader harmonized indicator set reinforces the need for multidimensional comparison. OECD Pensions at a Glance 2025 reports employment rates for ages 55–59, 60–64 and 65–69 and effective labor-market exit ages using common definitions, while Eurostat provides comparable healthy-life-years-at-65, adult-learning and digital-skills indicators. These domains are available for the eight Nordic–Baltic countries, but they should not be collapsed into a single table implying that employment, health, learning, digital skills and pension adequacy are measurements of one latent national construct. Instead, the framework treats them as harmonized indicators of distinct dimensions—participation, capacity, learning opportunity, digital resources and material security—that can be analyzed in parallel and linked to the propositions. Where a single source does not provide equivalent longitudinal microdata for all eight countries, the comparison should combine harmonized OECD/Eurostat indicators with national longitudinal or register evidence and report the coverage gap explicitly (Eurostat, 2026a,b; OECD, 2025b).

### 5.2 Institutional context: pension adequacy and the psychological meaning of retirement choice

Retirement institutions are treated here as contextual conditions that shape the psychological and organizational meaning of later-life employment. As shown in Table 2, we therefore summarize pension-age rules that actually apply in 2026 rather than modeled pension ages for future cohorts. The comparison also shows whether earlier or flexible pension access is possible, because the same employment status at older ages may reflect very different combinations of preference, work ability, job quality and financial constraint. This interpretation is particularly important where economic insecurity restricts the practical scope for retirement choice. Importantly, the countries are not fully comparable through a single ‘retirement age': Sweden has no fixed retirement age, Finland uses cohort-specific earnings-related pension ages, and Norway and Estonia permit substantial flexibility in pension withdrawal.

**Table 2:** Pension-age rules and retirement flexibility in 2026, grouped by Nordic and Baltic region.

Country	2026 pension-age reference	Early/flexible access relevant in 2026	Relevance for aging-at-work research
Nordic countries			
Denmark	67	State pension age is 67 for cohorts reaching pension age in 2026. Earlier pathways include voluntary early-retirement arrangements (efterløn) and senior pension for eligible groups; these routes differ from the statutory old-age pension.	The statutory age is cohort-specific, so age norms and retirement expectations differ between generations.
Finland	64 years 9 months	People born in 1961 reach their earnings-related old-age pension age at 64 years 9 months in 2026; later retirement increases pension.	A cohort-based retirement age makes late-career planning closely linked to age, work ability and incentives to continue.
Iceland	67	Old-age pension may start from 65 with a permanent actuarial reduction.	Flexible timing means that observed work after 65 cannot be interpreted as a uniform retirement decision.
Norway	67 (reference age)	Flexible old-age pension can generally be drawn from 62 if accrued pension is sufficient; work and pension can be combined.	The wide withdrawal window makes perceived choice and financial resources important for interpreting continued employment.
Sweden	No single fixed retirement age; riktålder 67 applies as the system reference age from 2026	Income and premium pension can be drawn earlier subject to birth cohort; for example, people born in 1961 can draw from 63. The 1959 cohort has a riktålder of 66.	Multiple age thresholds make retirement a gradual and highly individualized transition rather than a single statutory event.
Baltic countries			
Estonia	65	Flexible old-age pension can be taken up to 5 years earlier with sufficient pensionable service, with a permanent actuarial reduction; work and pension can be combined. Old-age pension may also be deferred, which increases the future pension.	Flexibility increases individual choice but also makes financial circumstances and employment quality central to retirement timing.
Latvia	65	Early old-age pension is available from 63 subject to qualifying conditions; before normal pension age, payment and employment restrictions apply.	The distinction between standard and early pension pathways is relevant to whether labor-market exit reflects health, constraints or preference.
Lithuania	65 (men and women)	Early old-age pension is available up to 5 years before the statutory pension age for individuals meeting the required pension insurance record and specified employment and income conditions.	The completed equalization of pension age provides a common age threshold, while early-access conditions and individual resources still shape actual retirement behavior.

Information applicable in 2026 was checked against identifiable national pension/social-insurance authorities: Denmark (borger.dk/Udbetaling Danmark), Finland (Finnish Center for Pensions), Iceland (Social Insurance Administration/island.is), Norway (NAV), Sweden (Pensionsmyndigheten), Estonia (Sotsiaalkindlustusamet), Latvia (VSAA) and Lithuania (Sodra), accessed September 2026. Pension-age references are not treated as a single harmonized “normal retirement age,” because national systems define eligibility and early-exit routes differently. Statutory ages should also be distinguished from effective labor-market exit ages (OECD, 2025b).

## 6 Five propositions for sustainable aging at work

### 6.1 Proposition 1: Institutional opportunities require workplace implementation

Institutional incentives affect work–retirement behavior through workplace implementation rather than directly. Pension rules may alter the feasibility or attractiveness of later retirement, but managers and organizations determine many proximal conditions—workload, scheduling, autonomy, training, task adaptation, phased retirement and access to occupational health. Policy implementation may therefore amplify or attenuate institutional incentives, and age-management practices can be unevenly applied across occupations and workers (Andersen et al., 2020). Proposition 1: The positive association between institutional opportunities for later or flexible retirement and voluntary continued employment (stay rather than push/stuck) will be stronger where workers have access to age-inclusive job redesign, autonomy, learning and flexible arrangements; this cross-level association will weaken where such workplace capacity is absent or unequally implemented (Carlstedt et al., 2024; Egdell et al., 2020; Jonsson et al., 2020, 2021).

### 6.2 Proposition 2: Work-ability/job-demand misalignment predicts sustainability outcomes

Employment at older ages is not a sufficient indicator of sustainable aging at work. We propose that the relationship between extended employment and subsequent health and psychological wellbeing depends on the alignment between workers' evolving work ability and job demands. Where demands exceed available capacity and adequate workplace resources or adaptations are absent, continued employment should be associated with less favorable health and wellbeing trajectories than where demands, resources and capacity remain aligned. Psychosocial demands, autonomy, support, physical workload and chronic health limitations can influence this alignment, while flexible working hours, ergonomic interventions and individualized arrangements may restore it. Longitudinal evidence indicates that individualized work accommodations can extend labor-market participation among workers with poor health (Jonsson et al., 2019), while systematic-review evidence suggests that the health consequences of extended working lives vary with job quality and opportunities to reduce or modify work (Baxter et al., 2021). Cross-sectional European evidence likewise associates flexible working hours and ergonomic adaptations with continued employment at older ages (Reine and Rajevska, 2024), and adverse psychosocial and physical working conditions have been linked to less favorable trajectories of health, working capacity and employment exit (Eyjólfsdóttir et al., 2025a,b). This proposition can be challenged empirically if misalignment between demands and capacity does not predict less favorable subsequent sustainability outcomes, or if workplace resources and adaptations do not modify that relationship.

### 6.3 Proposition 3: Digitalization is sustainable when resources keep pace with demands

Digitalization changes both job demands and job resources. It can reduce physical demands and expand flexibility, but it can also increase cognitive demands, monitoring and skill obsolescence. Proposition 3: The association between greater digitalization of work and sustainable continued employment will be positive when accessible training, digital self-efficacy and opportunities for task adaptation increase resources at least commensurately with new demands, but negative when digital demands increase without corresponding resources. The proposition is therefore falsifiable through the direction of longitudinal demand–resource interactions rather than the general claim that technology can have mixed effects (Komp, 2022; Sadauskaitė et al., 2025; Soitu et al., 2026; Sundstrup et al., 2022).

### 6.4 Proposition 4: Formal flexibility yields unequal substantive choice

Formal flexibility does not imply equal substantive capability to use it. Proposition 4: The association between access to flexible work/retirement arrangements and perceived choice will be weaker—and stuck or push mechanisms more prevalent—among workers with low pension wealth, poor health, physically demanding or precarious work, interrupted careers or substantial caring responsibilities. Consequently, the same formal flexibility can widen inequalities when the resources required to use it are unequally distributed. These inequalities are expected to intersect with gender, occupation/class, migration history and ageism (Grødem and Hippe, 2021; Nyberg et al., 2019; Scherger, 2021; Spasova et al., 2025).

### 6.5 Proposition 5: Choice conditions the intention–behavior relationship

Previous research links retirement intentions to work quality, including physical and psychosocial demands, social support, control and rewards (Kairys et al., 2024; Sadauskaitė and Kairys, 2023). Retirement intentions are proximal motivational states rather than sustainability outcomes. They translate preferences and perceived opportunities into behavior, while subjective age, perceived control and material feasibility may condition this translation. Proposition 5: Congruence between retirement intentions and subsequent work–retirement behavior will be stronger when perceived control and substantive alternatives are high; under financial constraint, age discrimination or restricted exit options, intentions will less reliably predict behavior and involuntary push/stuck trajectories will be more common. Subjective age is treated as a psychological resource/appraisal that may shape intentions and perceived feasibility, but the framework does not assume a universal direction of moderation without further evidence (Kairys et al., 2026; Vickerstaff and van der Horst, 2021).

## 7 A Nordic–Baltic research agenda

The framework suggests that Nordic–Baltic research should test sequences and configurations rather than isolated indicators. Table 3 summarizes potential measures, feasible data sources and analytical approaches for operationalizing the framework. Designs should distinguish distal antecedents from proximal appraisals and mechanisms, follow movement between full employment, modified work, work–pension combinations, alternative benefit pathways and retirement, and model feedback to health and wellbeing. Healthy-worker selection and reverse causality require explicit attention: people remaining employed are positively selected on health and employability, while employment itself may subsequently affect health. Disability, sickness and unemployment benefits should be modeled as alternative exit routes rather than treated as retirement. Gender, occupation/class, migration and minority status, caring responsibilities and precarity should be examined as intersecting stratifiers rather than appended moderators. One relevant cross-national source is the Survey of Health, Aging and Retirement in Europe (SHARE; Börsch-Supan et al., 2013), although its country coverage is not equivalent across the full Nordic–Baltic set.

**Table 3:** Operationalization and feasible comparative data strategy.

Framework element	Example measure	Level/time	Feasible data sources	Analytical approach
Health/work ability	Self-rated health; limitations; work ability	Individual; repeated	Survey of Health, Aging and Retirement in Europe (SHARE; Börsch-Supan et al., 2013) for participating countries; SLOSH/Y-SLOSH and national cohorts; EU-LFS/EU-SILC for harmonized indicators	Longitudinal models; selection adjustment
Job demands/resources	Physical/psychosocial demands; control; support	Workplace/individual	EWCS; national working-life cohorts	Multilevel interactions; trajectories
Alignment	Demand–ability and need–supply compatibility	Cross-level; repeated	Harmonized survey modules or linked national studies	Response surfaces; latent profiles; QCA; multilevel interactions
Intentions/choice	Retirement intention; perceived control; preferred vs. expected timing	Psychological; repeated	SHARE modules where available; national aging/work cohorts	Mediation/moderation; transition models
Transition mechanisms	Push/pull/stay/stuck/jump indicators	Individual × context	Purpose-built harmonized items; national cohorts	Latent classes/profiles; sequence analysis
Institutions	Pension age; replacement; flexible access; alternative benefits	Country/time	OECD; MISSOC; national authorities	Country-level moderators; policy discontinuities
Material resources	Income; pension adequacy; poverty risk	Household/country	EU-SILC; OECD	Cross-level interactions; distributional analyses
Digital/learning	Digital skills; training participation	Individual/country/time	Eurostat ICT and adult-learning datasets; EWCS	Interactions with age/occupation; trends
Coverage gap	Iceland and Norway are not covered by SHARE in the same way as core SHARE countries	Design constraint	Nordic registers/national cohorts plus harmonized aggregate sources	Triangulation rather than a single eight-country micro dataset

No single micro dataset provides identical longitudinal coverage of all 8 countries. The framework therefore supports coordinated comparative designs, triangulation of harmonized aggregate indicators with national longitudinal/register data, and explicit reporting of coverage gaps rather than assuming full-country harmonization.

## 8 Discussion

From a psychology-of-aging perspective, cross-national differences in later-life employment are most informative when they are used to examine how individual motivations and capacities interact with workplace conditions and contextual constraints. High employment at older ages cannot establish that workers experience autonomy, wellbeing or sustainable aging at work. Likewise, work after pension eligibility cannot be interpreted as either positive engagement or financial constraint without information on retirement intentions, subjective age, health, job quality, material resources and perceived choice. Iceland is informative in this respect: research on social disadvantage and poverty highlights the importance of economic resources (Guðmundsson et al., 2023), while studies of later-life work show that psychosocial job resources, work demands and health are linked to employment exit and trajectories around retirement (Eyjólfsdóttir et al., 2025a,b). The finding that both health and modifiable workplace adaptations are associated with continued employment among older adults further supports analyzing person–workplace alignment rather than treating age or pension eligibility as sufficient explanations (Reine and Rajevska, 2024). The Nordic–Baltic comparison therefore functions as a contextual framework for examining psychological and organizational mechanisms rather than as a ranking of pension systems.

The Sustainable Aging-at-Work Alignment Framework also clarifies why policy evaluation should not use employment rates as the sole outcome or benchmark. A rising older-worker employment rate may coexist with worsening health, financial pressure, involuntary postponement of retirement or widening inequalities (Phillipson, 2019; Scherger, 2021). Benchmarking countries solely on employment therefore risks rewarding the outcome of staying in work without distinguishing whether workers have the capability, job quality and economic freedom to do so on acceptable terms. Policy evaluation should also avoid underestimating the embedded nature of ageism. Age norms and stereotypes can affect not only retirement preferences but also opportunities and constraints in recruitment, retention, training, job redesign and access to flexible work. A reform can increase employment while worsening inequalities if workers in poor health are pushed toward alternative benefits or remain in unsuitable jobs; similarly, workplace and digital interventions may benefit some occupational groups while excluding others. Sustainability is consequently multidimensional, temporal and distributional, and must be evaluated in relation to both outcomes and the opportunities available to different groups (Vickerstaff and van der Horst, 2021).

Practical implications follow from specific mechanisms rather than from a general objective of retaining older workers. Where misalignment between work ability and demands generates push, ergonomic or psychosocial redesign and idiosyncratic arrangements target the mechanism directly. Where inadequate skills or digital self-efficacy create push, accessible learning and supported job crafting can restore resources. Where workers are stuck because retirement is financially inaccessible, workplace retention alone does not resolve the underlying lack of choice; pension adequacy and income protection become relevant. Where pull or jump reflects a genuinely preferred transition, retirement may itself be a sustainable outcome. Mid-life career reviews and transparent retirement conversations can improve perceived control and identify misalignment before it produces sickness absence or unwanted exit, but access and effects should be assessed across occupational and socioeconomic groups (Carlstedt et al., 2024; Egdell et al., 2020; Jonsson et al., 2020, 2021; Turek et al., 2020).

For research, the Nordic–Baltic region offers an opportunity to test whether similar outcomes arise through different pathways. Alignment can be operationalized through multilevel interactions, response-surface models for specific fit relations, latent profiles or configurational/QCA approaches, while work–retirement processes can be examined with event-history, multistate and sequence models. Analyses should address healthy-worker selection, reverse causality and alternative exit routes, and should distinguish intentions, decisions, behavior and subsequent consequences. Because SHARE does not provide equivalent coverage for the full eight-country set, a realistic strategy combines harmonized European indicators with coordinated national longitudinal/register studies rather than assuming a single fully harmonized microdataset.

## 9 Strengths and limitations

A principal strength of the analysis is its explicit separation of antecedents, alignment/misalignment, transition mechanisms, observed trajectories and sustainability outcomes. Successful aging at work refers here to the broader literature on adaptive functioning and maintenance of person–work fit across the lifespan (Kooij et al., 2020). Sustainable employability concerns the capability to achieve valued work outcomes now and in the future while safeguarding health and welfare (van der Klink et al., 2016). Work ability is an antecedent capacity relative to work demands, not a synonym for sustainability. Sustainable working lives is the broader policy and population-level domain. Sustainable aging at work, as used here, is specifically the temporal evaluation of late-career work–retirement trajectories in relation to health, wellbeing, autonomy and meaningful choice.

The article has limitations. It is a theory-building comparative synthesis, not a systematic review, and the evidence base remains uneven across countries and constructs. Aggregate indicators use different age ranges, denominators and institutional definitions and cannot establish individual voluntariness or causality. Healthy-worker selection and reverse causality complicate associations between health, job quality and continued employment, while disability, sickness and unemployment pathways may substitute for retirement differently across countries. Alignment is proposed as a dynamic cross-level construct rather than a validated scale: objective and perceived alignment may diverge, compensation among resources may be nonlinear, and push/pull/stay/stuck/jump mechanisms may overlap or change over time. Country cases are heuristic and should not be read as fixed welfare-regime classifications. The framework also requires further development regarding caring, migration/minority status and precarious employment. These limitations make falsification and comparative testing central to the research agenda rather than implying that the framework has already been empirically demonstrated. In addition, the published comparative summaries do not provide fully harmonized country-specific percentages for every retirement motive; where these are unavailable, the manuscript reports the limitation rather than imputing or inferring values.

## 10 Conclusion

The theoretical contribution of the revised Sustainable Aging-at-Work Alignment Framework is not the identification of another list of determinants but a process logic linking multilevel conditions to alignment/misalignment, transition mechanisms, trajectories and sustainability outcomes over time. It explains why the same observed outcome—continued work, work–pension combination or retirement—can reflect different configurations of capability, opportunity, constraint and choice. The Nordic–Baltic comparison supplies institutional variation with which to test these relationships, while ageism and socioeconomic inequalities shape access to both work and retirement alternatives. The framework therefore generates a research agenda in which continued employment, modified employment and retirement can each be sustainable or unsustainable depending on how they are produced and experienced. Policy success should consequently be evaluated not simply by how many people remain employed, but by whether people can remain, adapt work or leave when needed without avoidable deterioration in health, wellbeing, autonomy or substantive choice.

## Data Availability

This article draws on previously published literature and publicly available sources cited in the manuscript. No new primary data were collected. Further inquiries can be directed to the corresponding author.
